# Serpentine Overburden Products—Nature-Inspired Materials for Metal Detoxification in Industrially Polluted Soil

**DOI:** 10.3390/toxics11120957

**Published:** 2023-11-23

**Authors:** Marina V. Slukovskaya, Anna G. Petrova, Liubov A. Ivanova, Tatiana K. Ivanova, Irina A. Mosendz, Andrey I. Novikov, Anna A. Shirokaya, Mariia V. Kovorotniaia, Taras L. Panikorovskii, Irina P. Kremenetskaya

**Affiliations:** 1 Laboratory of Nature-Inspired Technologies and Environmental Safety of the Arctic Region, Kola Science Centre, Russian Academy of Sciences, 184209 Apatity, Russia; petrova_anna93@mail.ru (A.G.P.); tk.ivanova@ksc.ru (T.K.I.); ia.mosendz@ksc.ru (I.A.M.); t.panikorovskii@ksc.ru (T.L.P.); 2 I.V. Tananaev Institute of Chemistry and Technology of Rare Elements and Mineral Raw Materials, Kola Science Centre, Russian Academy of Sciences, 184209 Apatity, Russia; a.novikov@ksc.ru (A.I.N.); a.shirokaia@ksc.ru (A.A.S.); i.kremenetskaia@ksc.ru (I.P.K.); 3 Institute of Biology, Ecology, and Agrotechnology, Petrozavodsk State University, 185000 Petrozavodsk, Russia; 4 N.A. Avrorin Polar-Alpine Botanical Garden-Institute, Russian Academy of Sciences, 184209 Apatity, Russia; ivanova_la@inbox.ru; 5 LLC. Eco-Express Service, 195112 Saint-Petersburg, Russia; kremenmasha@gmail.com

**Keywords:** eco-friendly materials, serpentine minerals, nickel, copper, industrially polluted peat, soil mixtures, geochemical barriers, geochemical migration, non-metric multidimensional scaling (NMDS) method

## Abstract

The possibility of plants growing on serpentine soils and the ability of serpentine minerals to accumulate significant amounts of metals was the basis for developing a method for using serpentine-containing materials to restore vegetation in areas with a high level of metal pollution. Serpentine-containing products obtained from phlogopite mining overburden (Kovdor, Murmansk region, Russia) with and without thermal activation were used in a field experiment on the remediation of industrially polluted peat soil. According to the geochemical mobility of the components, one of four fractions was allocated depending on the acidic (HCl) concentration of the solution used for the material treatment: readily mobile (0.001 mol/L), mobile (0.01 mol/L), potentially mobile (0.1 mol/L), and acid-soluble (1.0 mol/L). This study showed that the addition of serpentinites to peat soil changed the fraction composition. The most significant changes were noted for serpentinite components such as Ca and Mg: their concentrations increased 2–3 times even in the smallest portion of serpentine material. On the contrary, the contents of metals in the readily mobile fraction decreased 3–18, 3–23, 5–26, and 2–42 times for Cu, Ni, Fe, and Al, respectively. The main factor causing the decrease in metal mobility was the pH rise due to the release of Ca and Mg compounds into the soil solution. This study showed that the addition of serpentine-containing material at 25 vol.% to peat soil was sufficient to create a geochemical barrier with a stable-functioning vegetation cover. All serpentine-containing materials are recommended for the remediation of large industrially polluted areas.

## 1. Introduction

Serpentine minerals are widely distributed in the Earth’s crust, forming serpentine provinces with specific vegetation in some regions [1]. Serpentinites are found in different places in the world, with global reserves estimated at hundreds of millions of tons. The most important serpentine deposits are in Australia, Armenia, Italy, Russia, the USA, Canada, Paraguay, New Zealand, and Brazil [2].

Serpentinites are associated with tectonic blocks and the intrusion of ultramafic rocks, and their sedimentary and regionally metamorphosed derivatives. The soil cover of these sites is characterized by specific properties such as (i) a low Ca/Mg ratio, causing the inhibition of Ca uptake; (ii) the toxic effect of high Mg levels; (iii) low Ca levels; (iv) the toxic effects of large concentrations of heavy metals, particularly Ni; (v) low levels of available Fe due to high pH values and competition with Ni and Co; (vi) low levels of the available macronutrients N, K, and P; (vii) drought due to soil shallowness, a sandy texture, and high erodability due to the sparse vegetation; and (viii) a dark color and consequently high temperature and drought [1,3].

Serpentine soils have elevated amounts of trace metals, such as nickel, cobalt, and chromium, in comparison to non-serpentine soils [4,5,6]. These soils can absorb heavy metals not only from natural or lithological sources but also from industrial activities, which leads to an increase in their concentrations in a mobile form [7]. At the same time, together with clays, serpentine minerals can be attributed to eco-friendly materials [8,9,10]. The sorption of metals by a serpentine mineral can occur via the metal entering the structure of the mineral [11,12,13,14], adsorption on the mineral’s surface [2,15], and the precipitation of poorly soluble compounds in an alkaline medium [16]. These processes can be intensified by modifying serpentines, namely, via heat treatment. During the thermal decomposition of a serpentine, the mineral is partially dehydroxylated. This process is accompanied by the disordering of the silicate layer and iron oxidation from Fe^2+^ to Fe^3+^; as a result, the serpentine passes into an amorphous unstable state [17,18]. The evident result of ameliorative measures using thermoactivated serpentines was the sustainable functioning of artificial communities of cereals with high productivity, growing steadily for 10 years in conditions of ongoing technogenic pollution at the experimental site [19].

Plants growing in serpentine soils form a special type of community: serpentine flora [20,21]. Plants of the nickel flora are the most widespread, some of them belonging to the category of hyperaccumulators. Hyperaccumulators of potentially toxic metals in serpentine soils are widespread everywhere (e.g., in Turkey, Greece, India, and Pakistan) [22,23,24], including the northern regions of the Russian Federation, for example, the Polar Urals and Chukotka [25].

Currently, the study of the mobility and bioavailability of elements (for example, potentially toxic metals) involves their extraction via the sequential treatment of one soil sample using nitric or hydrochloric acid with an increasing concentration. This procedure enables the isolation of elemental fractions from their most mobile forms to be firmly bound by the solid soil phases [26,27]. In addition, this method makes it possible to identify associations between elements in various geochemical fractions [28]. In an environmental risk assessment, it is more important to obtain information about the amount of potentially mobile fractions of chemical elements than to identify their exact chemical species [29]. The results obtained with the sequential extraction method can also be discussed in a qualitative way by comparing the chemical element distributions in different soil samples. In the current study, this method was used to compare serpentine sites in terms of creating geochemical barriers in areas with a high content of metal compounds.

The possibility of plants growing on serpentine soils and the ability of serpentine minerals to accumulate significant amounts of metals (especially nickel) was the basis for developing a method of using serpentine-containing materials to restore vegetation in the subarctic zone in areas with a high level of metal pollution, primarily of copper and nickel [30]. In long-term field experiments, different serpentine-containing products from serpentinite magnesite overburden (Khalilovo magnesite deposit, Orenburg region, Russian Federation), the lizardite-containing waste of olivinite mining (Khabozero olivinite deposit, Murmansk region, Russia), and the overburden of phlogopite mining (Kovdor phlogopite deposit, Murmansk region, Russian Federation) were used [19,31].

A long-term field experiment was conducted on materials with a wide range of serpentine mineral contents, which were derived from phlogopite overburden. The novelty of this study is the analysis of the effect of introducing different proportions of serpentine-containing materials into industrially polluted soil on the geochemical migration and transformation of chemical elements in soil mixtures.

The purpose of this study was to determine the effectiveness of the introduction of serpentine geochemical barriers in reducing the mobility of potentially toxic metals in the impact zone of an operating Subarctic copper and nickel enterprise. The practical task was to select the optimal dose and variety of serpentine-containing material for remediating large areas.

## 2. Materials and Methods

### 2.1. Materials

**Figure 1:**
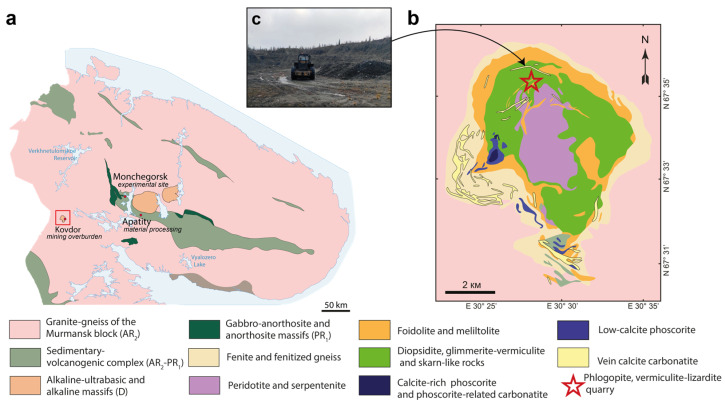
Locations of experimental sites of industrial barren land and the source of serpentine materials: (**a**) geological scheme of Kola Peninsula; (**b**) simplified geological map of the Kovdor massif (after Afanasyev and Pan’shin, with modification from [33]); reprinted from [32] under the Creative Commons Attribution (CC BY) license 4.0; (**c**) phlogopite quarry (photo by authors).

Serpentine-containing mineral materials from the Kovdor phlogopite deposit were used in the experiment. The Kovdor massif of peridotites, phoidolites–melilitolites, phoscorites–carbonatites, and associated metasomatic rocks (phenites, diopsidites, phlogopitites, and skarnoids) is located in the southwest part of the Murmansk region (67°33′ N, 30°31′ E) and is a polyphase intrusion (Figure 1), which intrudes about 380 Ma into the Archean granite–gneisses of the Belomorsky block [32]. Rocks of the vermiculite complex occur within a 200–400 m wide band, elongated north–south in an area of hydrothermally altered olivinite, including diopside–phlogopite rocks, from near the surface to depths of 50–70 m. This band is associated with schistose zones containing numerous ijolite dykes. The phlogopite beyond these zones is almost unaltered in all rock types (except for a slight decrease in potassium content compared with theoretical values), which refers to phlogopite–pyroxene rocks, phoscorites, foidolites, rocks of the phlogopite complex, and host gneiss. The vermiculite complex is a unique exception because it formed as a product of hydrothermal alteration of the diopside–phlogopite rocks formed after olivinite [33].

**Figure 2:**
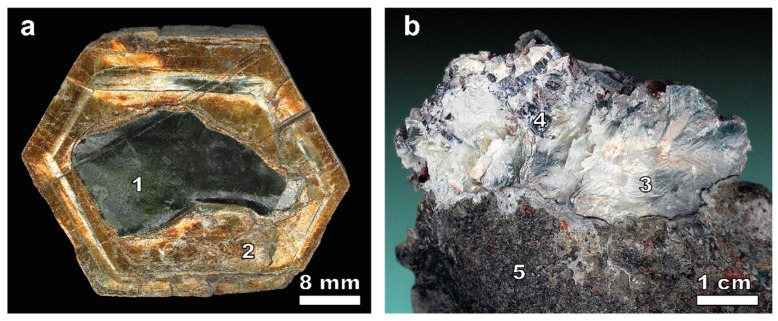
Phlogopite (1) relics in the core of hexagonal-shaped plates of vermiculite (2) (**a**). Lizardite-1T (3) pseudomorph after phlogopite with annite (4) from serpentinized forsterite–diopside–phlogopite rock (5) from the vermiculite complex rocks of Kovdor massif (**b**).

The hydrothermal alteration of diopside–phlogopite rocks has produced friable, brown rocks (color produced via goethite formation after magnetite) mostly composed of lizardite formed after forsterite or diopside, enclosing large (up to 1.5 m) golden-yellow, hexagonally shaped plates of vermiculite with phlogopite relics in the cores of crystals (Figure 2). The vermiculite is replaced layer by layer with lizardite. The associated minerals may include saponite, clinochlore, calcite, and goethite. The relics contain forsterite, diopside, hydroxilapatite, magnetite, and rare grains of richterite, nepheline, and åkermanite [34].

The vermiculite–lizardite overburden from the open mining of phlogopite (LLC Kovdorslyuda, Kovdor, Murmansk region, NW of Russian Federation) covers an area of more than 10 hectares and contains hundreds of tonnes of vermiculite and lizardite [31]. In a long-term field experiment on industrial barren land, which was heavily polluted with toxic metals, this waste was found to be an effective material for ecosystem initialization and primary soil formation [19,35]. To separate waste and obtain materials with increased efficiency, a technological scheme was developed for waste remediation via the production of serpentine-containing materials with different contents of vermiculite and lizardite [31].

Two mineral materials were used in the current study: the vermiculite–lizardite granular product, contained 25 wt.% lizardite and 37 wt.% vermiculite, and pyroxene product (PR), contained 8 wt.% lizardite and 5 wt.% vermiculiteVermiculite-lizardite product was used as the initial material (VL) and after firing at 500 °C in a modular trigger furnace designed by A.I. Nizhegorodov (LT) [36].

### 2.2. Design of Field Experiment

**Figure 3:**
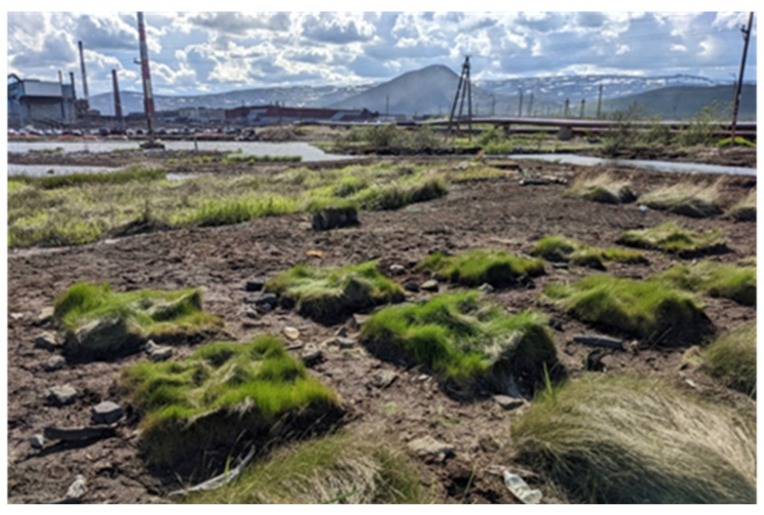
Experimental site.

A field experiment with serpentine-containing materials was started in July 2020 on an experimental site with peat soil (PT) highly polluted with Cu and Ni due to the long-term aerial emissions of a nonferrous plant (Monchegorsk site). The field experiment site is shown in Figure 3.

Soil mixtures were directly created in the zone impacted by the Cu/Ni plant; for this, the surface layer of the peat soil (0–10 cm) was mechanically homogenized and mixed with mineral material from the mining waste. The experiment included three series. In the VL and PR series, the soil mixtures were formed in proportions of 25/75, 50/50, and 75/25 (by volume); the control was 100% mineral material. In the LT series, the share of the mineral material was 1, 5, or 10 vol.% due to the high alkalinity of the material. The area of each mixture was 1–2 m^2^. The vegetation cover was created from preliminarily germinated grass seeds of *Festuca arundinacea* Schreb. in 1 cm layers of expanded vermiculite.

### 2.3. Methods

#### 2.3.1. Chemical Analyses

Soil mixtures and peat soil (*n* = 3) were sampled on an experimental site of industrial barren land near a Cu/Ni plant from depths of 0–10 cm in September 2022, at the end of the third growing season. Samples were air-dried and homogenized through a 2 mm sieve. The acidity of the soil suspensions was determined using an I-160M ionomer in a ratio of 10 g of soil per 100 mL of deionized water after incubation for 1 h.

Fractional analysis of the soil mixtures was performed through sequentially treating the soil samples with hydrochloric acid solutions with gradually increasing concentrations: 0.001, 0.01, 0.1, and 1.0 mol/L. An acid solution (100 mL) was poured into a 10 g soil sample and kept for 24 h; then, the solution was separated from the solid phase via centrifugation for 30 min at 4000 rpm. The resulting solution was passed through a MFAS-OS-2 membrane filter (Vladipor manufacture, Vladimir, Russia) with a pore size of 0.45 μm and analyzed using atomic emission spectrometry on an ICPE-9000 instrument (Shimadzu, Tokyo, Japan). The sediment from the filter was combined with a soil sample and washed with an acid solution into a centrifuge tube.

The operating mode was set in accordance with the manufacturer’s recommendations: solvent rinsing time, 10 s; sample washing time, 60 s; holding time, 30 s; number of measurements, 2; high-frequency generator output power, 1.2 kW; carrier gas consumption, 0.7 L∙min^−1^; additional gas consumption, 0.6 L∙min^−1^; plasma gas flow, 10 L∙min^−1^; measurement mode, highly sensitive (H); calibration graph mode, background correction method. Quality was controlled by analyzing the standard samples CRM-SOIL-A (Certified Reference Material Soil Solution A) and CWW-TM-A (Certified Waste Water-Trace metals Solution A). For all samples, the standard error did not exceed 5%.

#### 2.3.2. Interpretation of the Sequential Fractioning Results

According to the geochemical mobility of the components, four different fractions were distinguished depending on the concentration of the acid solution used for the material treatment: readily mobile (0.001 mol/L), mobile (0.01 mol/L), potentially mobile (0.1 mol/L), or acid-soluble (1 mol/L) (Table 1). The sum of all fractions corresponds to the conditionally total content, which many authors have recommended considering when analyzing the chemical contamination of soil samples. Table 1 presents the scheme followed for processing the fractionation results.

### 2.4. Powder X-ray Diffraction

Powder X-ray diffraction data were collected using a Rigaku Mini Flex 600 diffractometer in the 2θ range of 5–70° (Cu*K*α; 1.5418 Å) with a scanning step of 0.02° in 2θ and a rotation speed of 20 rpm. A normal-focus Cu X-ray tube was operated at 40 kV and 15 mA. The experimental curves were integrated using the SmartLab Studio II program [37]. The Voigt profile was used for the approximation of experimental curves.

### 2.5. Statistical Analysis

Statistical processing, including the ordination of the samples, was performed using the R programming language in the RStudio development environment (packages vegan, dplyr, plyr, car, and ggplot2). For ordination, we used the nonmetric multidimensional scaling (NMDS) method [38,39,40,41,42].

## 3. Results

### 3.1. Characteristics of Serpentine-Containing Soil Mixtures

**Figure 4:**
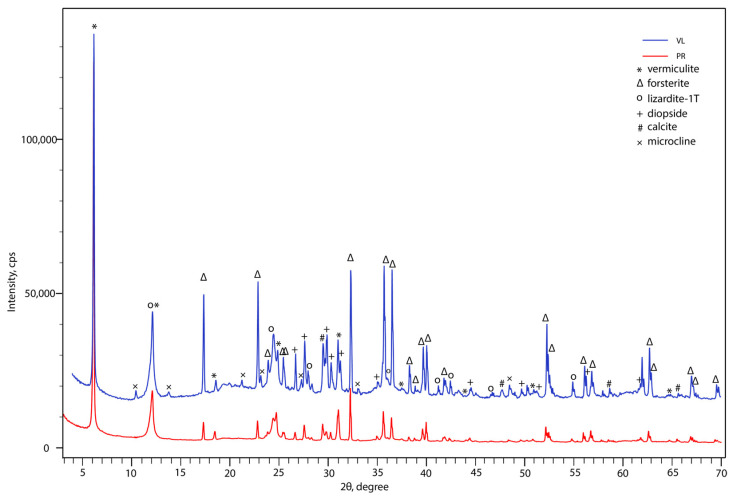
Powder XRD pattern of pyroxenite product (PR—red curve) and vermiculite–lizardite product (VL—blue curve).

The mineral composition of the initial serpentine-containing materials is shown on Figure 4. The most intensive reflections relate to vermiculite (No. 00-060-0341), as indicated by the series of reflections with *d*(Å): 14.38, 7.30, 4.80, 3.60, 2.88, and 2.0. Weak diagnostic lizardite-1T (No. 00-050-1625) reflections, with *d*(Å): 7.30,3.89, 3.65, 2.42, 2.14, and 1.42, are also present in the pattern. The third main component of this mixture is forsterite (No. 01-073-6338), which presented with numerous reflections: 5.12, 3.89, 3.73, 2.77, 2.57, 2.46, 2.35, 2.32, 2.27, 2.25, 2.16, 2.03, 1.88, 1.79, 1.74, 1.64, 1.62, 1.59, 1.57, 1.51, 1.50, 1.48, 1.37, and 1.35. Th presence of the reflections 3.65, 3.34, 3.23, 2.99, 2.95, 2.57, 2.52, 2.15, 2.13, 2.11, 2.04, 2.02, 1.84, 1.83, 1.82, 1.80, 1.75, 1.72, and 1.67 could be attributed to diopside (No. 01-075-1092). A minor admixture of calcite (No. 01-086-2335) was also detected through the reflections at 3.03, 2.49, 2.09, and 1.87. The vermiculite–lizardite product contained a microcline (No. 01-087-1788) admixture, as indicated by reflections with *d*(Å): 6.43, 4.19, 3.65, 3.59, 3.34, 3.27, 3.24, 2.96, 2.90, 2.89, 2.53, 2.49, 2.09, 1.91, 1.78, 1.72, 1.52, 1.42, and 1.41.

The physical and chemical characteristics of the materials used in the three series of field experiments on the remediation of industrial barren land are presented in Table 2 and Table 3.

Table 4 presents the physical and chemical parameters of the soil samples taken at the experimental sites. The sites substantially differed in density, from 0.29 kg/dm^3^ for the original peat to 1.69 kg/dm^3^ for the pyroxene product without peat addition. Field moisture also varied depending on the composition of the soil mixture. The addition of serpentine minerals reduced the actual acidity, while the redox conditions changed insignificantly. Together, pH/Eh conditions contribute to a decrease in the geochemical mobility of nickel [43]. The irregular (not directly proportional to the share of mineral materials) changes in the characteristics of the soil mixtures reflected their natural heterogeneity.

### 3.2. Composition of Geochemical Fractions of Serpentine-Containing Soil Mixtures

#### 3.2.1. Influence of Mineral Composition and Share of Serpentine-Containing Materials in Soil Mixture on the Distribution of Chemical Elements by Fraction

**Figure 5:**
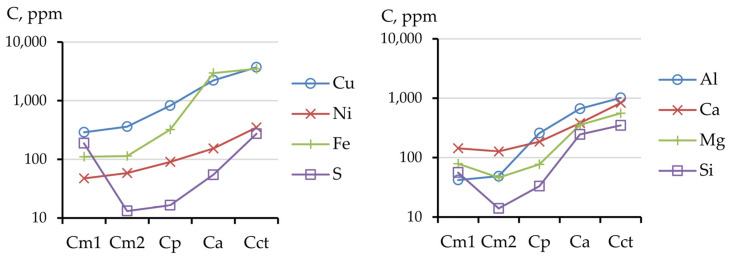
Content of elements in different fractions in industrially polluted peat soil.

According to the results of the treatment of industrially polluted peat soil with a weak solution of HCl (0.001 mol/L), the most mobile elements were copper and sulfur; calcium and iron leached in lower concentrations (Figure 5). We assumed that copper sulfate, as well as calcium and iron carbonates, went into this fraction. Nickel dissolved slightly less than magnesium and silicon under these conditions.

The solubility of nickel increased with the increasing HCl concentration (up to 0.01 mol/L), while the amount sulfur in the leaching solution considerably decreased. After treating the peat soil with a solution of 0.1 N HCl, Cu remained, in terms of mobility, followed by Fe, Al, Ca, and Ni; Mg and Si directly followed Ni in this order. In the 1 mol/L HCl extract, the order of the elements was generally the same, and only Fe and Cu, as well as the group of Mg, Si, and Ni, changed their ranking. The metal compounds that were extracted with 0.1 and 1 mol/L solutions likely had a similar chemical composition and only their resistance to acid solutions differed.

All fractions contained only small amounts of Na, P, and Mn, which is why these elements were excluded from further consideration. Potassium was also not considered as it was added into the soil mixtures as part of a complex NPK fertilizer and does not affect the geochemical migration of metals.

Figure 5 depicts a diagram for the entire set of elements considered in this study in all the fractions obtained during the processing of the initial polluted peat soil. An increase in the acid concentration for all elements, except S and Si, corresponded to an increase in the extraction of those components into the solution. Sulfur was mainly contained in the most mobile (readily mobile) fraction, and silicon was evenly distributed over all mobile fractions. The highest silicon content was observed in the inert fraction, as were other petrogenic elements: iron and aluminum.

**Figure 6:**
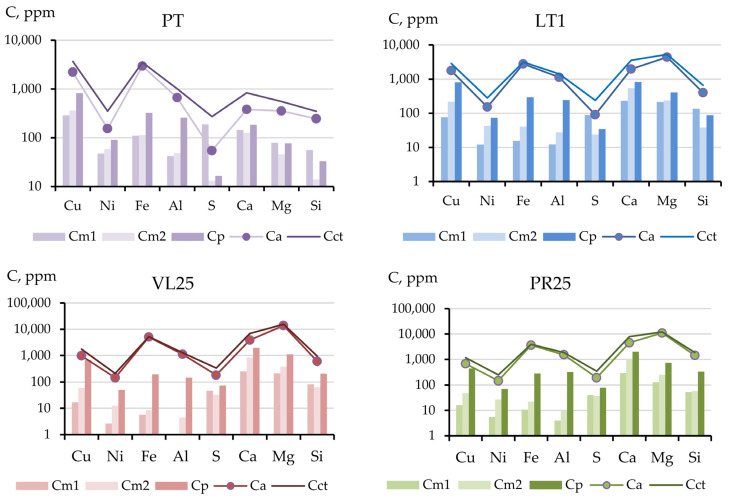
Fractional composition of industrially polluted peat soil and soil mixtures of peat and serpentine-containing materials; labels provided in Table 4.

**Figure 7:**
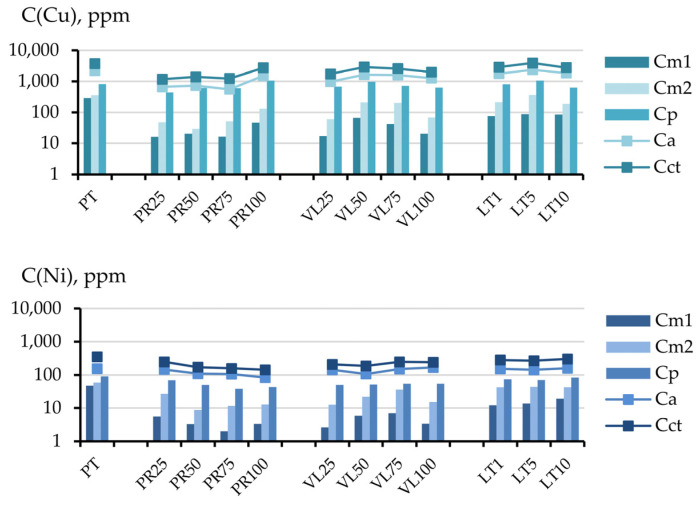

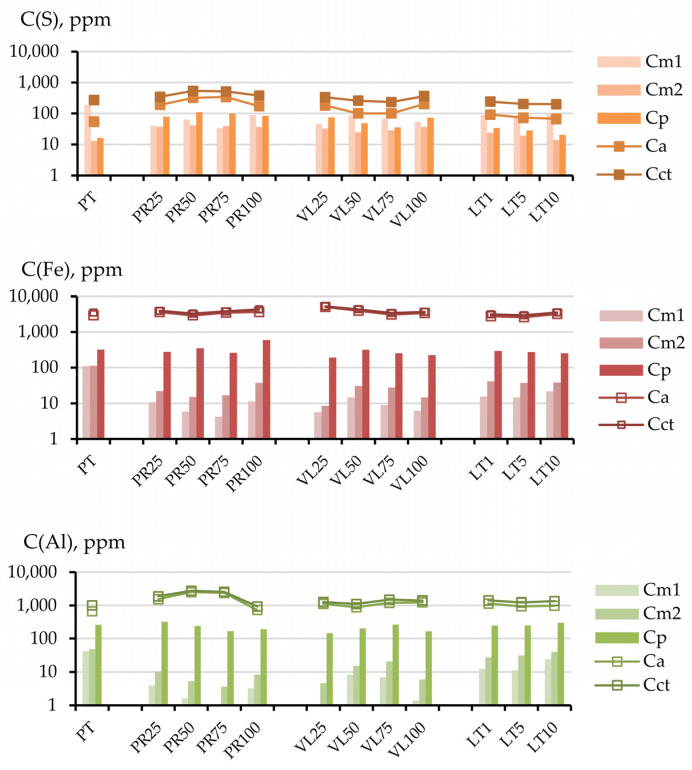
Content of element fractions in soil mixtures of peat and serpentine-containing materials.

The addition of serpentinites to the peat soil dramatically changed the fraction composition (Figure 6). Even the addition of a small amount of thermally activated serpentinite (1 vol.%) to the LT1 mixture shifted the contents of the components in various fractions. The most remarkable changes were noted for the serpentinite calcium and magnesium components: their concentrations increased 2–3 times. However, the contents of the metals in the mobile fractions decreased several times, i.e., the content of the readily mobile fraction decreased 3–18, 3–23, 5–26, and 2–42 times for Cu, Ni, Fe, and Al, respectively (Figure 7). Unlike the metals, the amount of sulfur increased in the mobile fractions and in the conditionally total content.

#### 3.2.2. Influence of the Dilution Factor of Technogenic Peat with Serpentine-Containing Materials on the Contents of the Cu, Ni, and S Fractions

**Figure 8:**
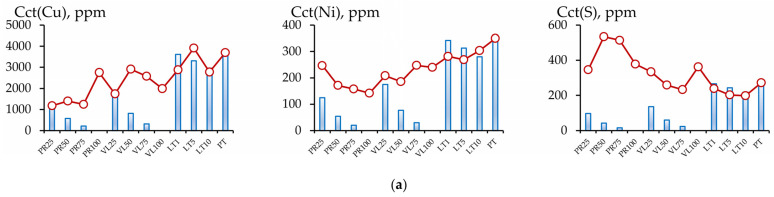

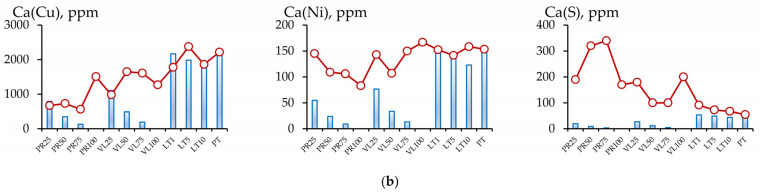
Comparison of experimental (line with markers) and calculated values (histogram) of the contents of components in the conditional total (**a**) and acid-soluble (**b**) fractions of the experimental soil mixtures.

**Figure 9:**
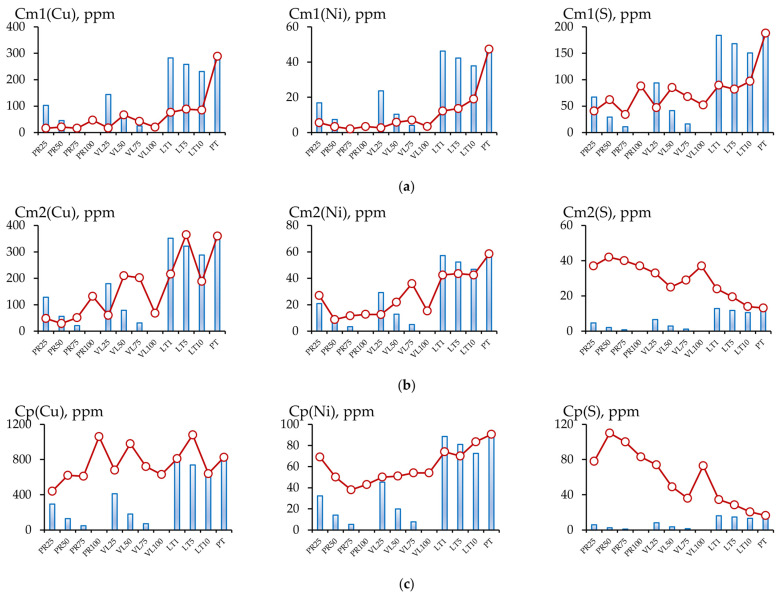
Comparison of the experimental (line with markers) and calculated values (histogram) of the contents of various components in the readily mobile (**a**), mobile (**b**) and potentially mobile (**c**) fractions.

The change in the content of the various forms of the components in the soil could have been the result of both the influence of serpentinites and the dilution of the peat substrate in the soil mixture. Figure 8 and Figure 9 compare the chemical contents of the soil mixtures and the data calculated based on the elements’ concentrations in the initial peat (in the corresponding fractions) and the share of peat in the soil mixture.

A substantial accumulation of sulfur in the conditional total fraction under field conditions was observed in the PR and VL series. This 2–10 times exceeded the calculated values, and the accumulation mainly occurred in the inert fraction extracted using the 1 mol/L HCl solution. A minimal accumulation of sulfur was observed in the readily mobile fraction in the PR and VL series, with a serpentinite content of 50–100 vol.%. In other soil mixtures in the experiment, a decrease in the concentration of the most mobile sulfur was observed.

The nickel content in the conditional total fraction in all soil mixtures in the experiments with non-activated serpentinites PT and VL also exceeded the calculated values. The copper contents in experiments PT25 and VL25 were lower than the calculated values. The same tendencies were observed for the inert fraction. For copper, nickel, and sulfur, the inert fraction contributed the most to the conditional total content.

#### 3.2.3. The Migration Coefficients of Pollutants

**Figure 10:**
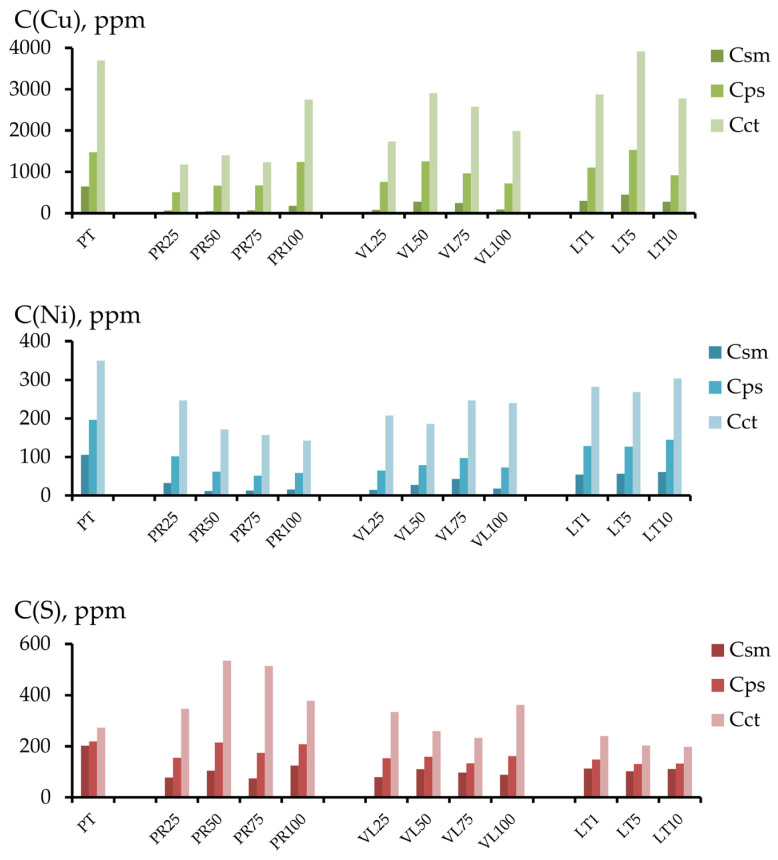
The content of elements in different migration fractions.

A migration coefficient was proposed to evaluate the geochemical mobility of pollutants based on the data on the ratio of element concentrations in various fractions [44]. Figure 10 presents the contents of the components in the mobile fractions as the sum of short-term and potentially mobile fractions, as well as data for the acid-soluble fraction (Ca). In most of the experimental soil mixtures, the content of the mobile fractions in the soil mixtures were lower than in the initial peat soil; for nickel, this process was the most pronounced.

**Figure 11:**
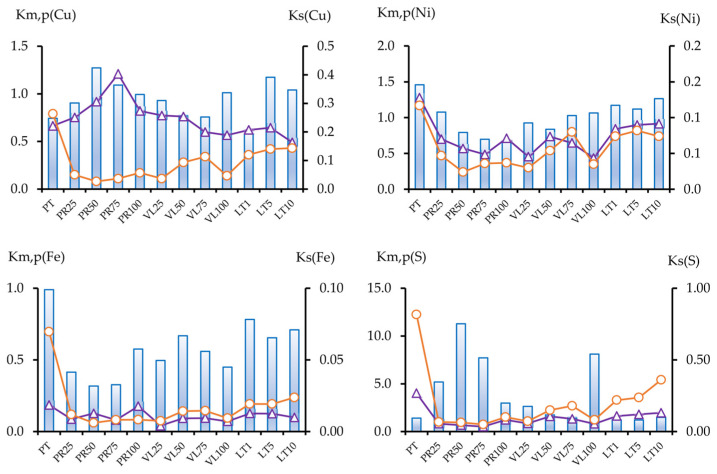
Short-term (Km) (○) and potential (Kp) (∆) migration coefficients and total migration coefficient (Ks) (histogram) of soil samples.

The determinations of the three mobile metal fractions showed that the content of the potentially mobile fraction varied within a narrow range of values for all soil mixtures, whereas the contents of Cm1 and Cm2 were substantially different in experiments with serpentinites compared with those in the initial technogenic peat soil (Figure 7 and Figure 10). The ratio of the sum of the readily mobile and mobile fractions to the potentially mobile fraction is called the coefficient of the short-term process of geochemical migration, Km (Figure 11). In the long term, the content of the components in the acid-soluble fraction may change due to the formation of new, less stable compounds under the influence of external factors, such as intake from the air and soil, cryogenic weathering, and alternating wetting and drying.

The ratio between the sum of the mobile fractions and the acid-soluble fraction (the coefficient of potential migration, Kp) is also an indicator of the geochemical mobility of the pollutants (Figure 11). The data are presented for the most notable pollutants in the study area: sulfur, copper, and nickel, as well as iron, which is an element that strongly impacts the geochemical status of metals. This indicator decreased in all serpentine-containing samples, compared with the initial peat soil, for Ni, Fe, and S, and increased for Cu in the PR series. Notably, the higher migration coefficient of copper compared with nickel is characteristic of serpentine soils [3]. This conclusion is consistent with those of another study that investigated increased Cu solubility in alkaline soils [45].

For copper, the same pattern for the third coefficient, the ratio of the conditionally total content to the total concentration (the total migration coefficient, Ks), was observed (Figure 10). Like copper, the Ks for sulfur was higher in the PR series, whereas a marked decrease in the Ks for iron was noted.

**Figure 12:**
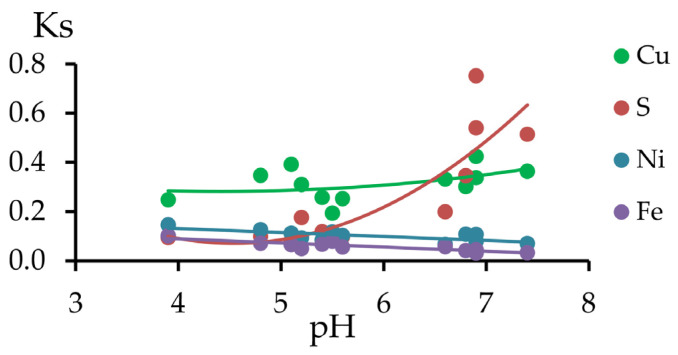
Relationship between the total migration coefficient and pH of soil mixtures.

The relationship between the Ks and the values of the actual acidity of the soil mixtures is shown in Figure 12. As the pH increased, the total migration coefficient Ks decreased for Ni and Fe and increased for Cu and especially for S.

From the results of the data analysis, we propose the following scheme: iron and copper sulfides in the polluted peat are present in the inert fraction, but under the influence of hypergenesis factors, they are oxidized with the formation of soluble sulfates, which, in turn, are fixed in the form of copper and iron hydroxo compounds under the alkaline conditions in serpentinite-containing soil mixtures. More alkaline conditions formed in the PR series, and higher Ks values for copper and sulfur were also observed compared with those of the initial peat soil. The short-term mobility of all elements decreased, the potential mobility increased for copper, and the total migration coefficient increased for copper and sulfur.

#### 3.2.4. Provision of Soil Mixtures with Alkaline Components and Silicon

**Figure 13:**
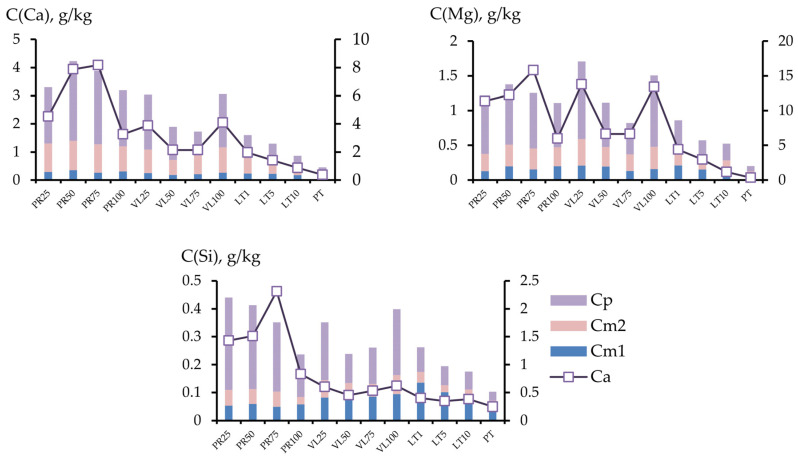
Ratio of the migration forms of components: Cm1, Cm2, Cp—left axis; Ca—right axis.

Copper, as a rule, is fixed on alkaline barriers, whereas nickel forms poorly soluble silicates [46]. The fractional composition of Si, Ca, and Mg in the soil mixtures is shown in Figure 13. The sum of the mobile calcium fractions in the PR series varied in the range of 3.2–4.2 g/kg, and 1.8 to 3.1 g/kg in the VL series, and was less than 1 g/kg in the LT series. The distribution of mobile magnesium and silicon in the PR and VL series was more uniform. The content of the acid-soluble fraction contrasted that of calcium and silicon; the PR series could be distinguished according to the contents of these elements.

**Figure 14:**
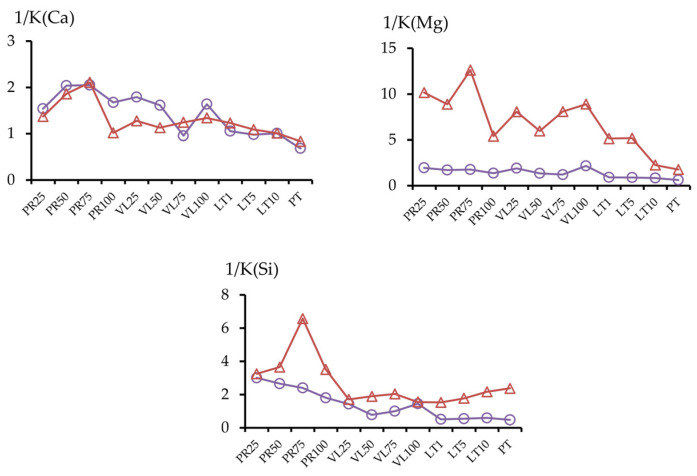
Inversed values of the coefficients of short-term (○) and potential migration (∆) for various elements.

To assess the supply of calcium, magnesium, and silicon compounds to various fractions, the reciprocal of the migration coefficient was used (Figure 14). The coefficients of the short-term (1/Km) and potential (1/Kp) supply of soil mixtures were close for calcium and silicon, whereas magnesium was more present in its potentially active form. The 1/Km values for magnesium varied in a narrow range of 0–2; for calcium, 1/Kp was in the same range; for silicon, this value in most soil mixtures varied in the range of 1.8–4, except for in PR75 (1/Kp = 6.3). The potential supply of magnesium was considerably higher; in soils with non-activated serpentinites 1/Kp varied from 5.1 to 12.4. The highest index was noted at site PR75.

Despite the high gross content of magnesium compared with calcium, the element concentrations differed by more than one order of magnitude. The Ca content in the mobile fractions was higher than the Mg content, whereas the opposite was revealed to be the case for the acid-soluble fraction. The higher content of the mobile Ca fraction may be due to Mg not preventing Ca absorption by plants in serpentine soil [47].

## 4. Discussion

**Figure 15:**
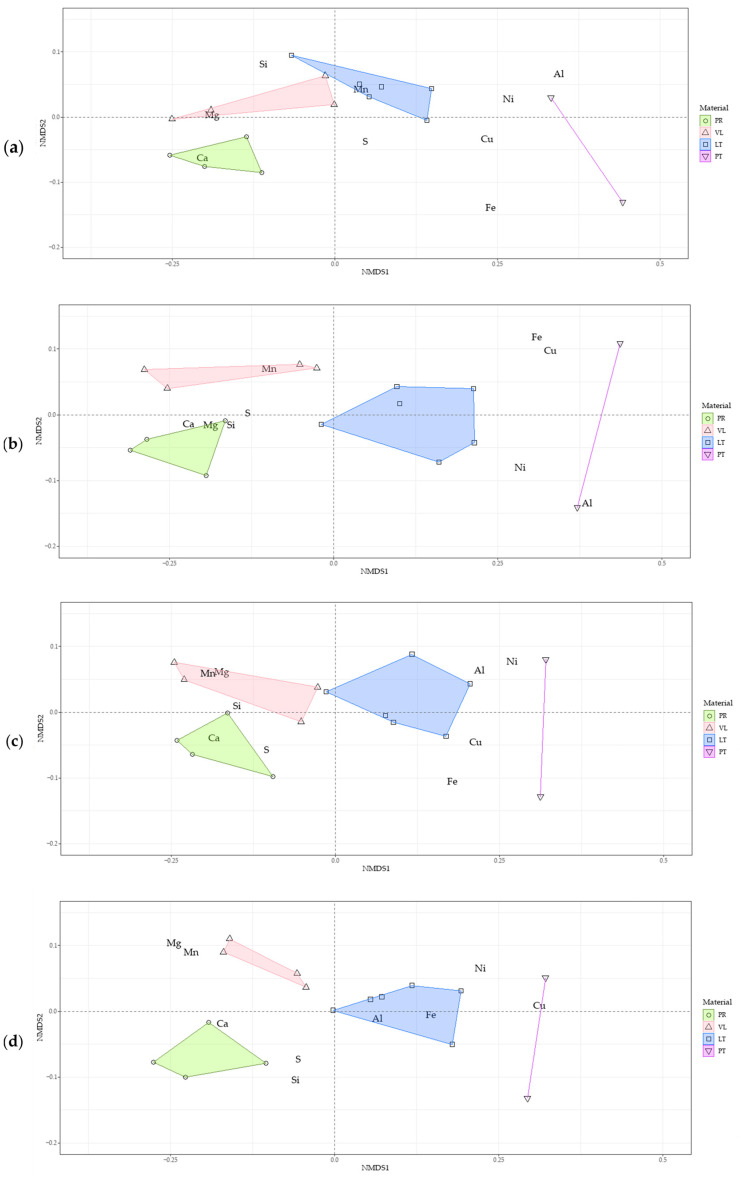
Ordination diagrams of the results of fractionation of soil samples: different fractions Cm1 (**a**), Cm2 (**b**), Cp (**c**), and Ca (**d**).

The fractional analysis data were processed using mathematical statistics methods [48]; the ordination of the samples is shown in Figure 15. The general trend for all fractions was the isolation of a group of metals (Cu, Ni, Fe, and Al). Samples of the LT series and the original peat were confined to one area. Another isolated group was Ca, Mg, and Si—the main components of serpentine materials. Manganese and sulfur were in an intermediate position between two groups of elements mentioned above. The samples in the PR series tend toward the calcium group, whereas the VL samples were divided between the PR and LT soil mixtures. Thus, based on these data, successive series of both experimental options (PT, LT, VL, PR) and groups of elements: (Cu, Ni, Fe, Al), (Mn, S), and (Ca, Mg, Si) were compiled.

In the PT–LT–VL–PR series, a gradual increase in pH was observed. In the row of elements, an increase was noted in the content of elements capable of displacing metals from phases that are metal sorbents (i.e., organic matter and Fe/Mn oxyhydroxides). Thus, based on the ordination diagrams, we drew a hypothesis about the nature of the main factors affecting the considered systems: this occurs as the result of a change in the acid–base properties and the displacement of metals from the phases depositing copper and nickel, which are the main pollutants in industrial peat soil.

In our long-term field experiment, the addition of initial and thermally activated serpentinites to highly industrially polluted peat soil decreased the short-term geochemical mobility (and, therefore, the toxic effect) of metal pollutants. Based on the results of NMDS data analysis, we argue that the main factor causing the decrease in short-term mobility was the increase in pH because of the release of calcium and magnesium compounds into the soil solution. The change in total migration ability depended on the increased element species, copper, and especially sulfur contents in the soil mixtures with the addition of serpentinites, in comparison with those in the initial peat soil.

Studies are required of the interactions between the mineral materials and soil phases responsible for the dynamic pools of metals under different acid–base conditions, as they pose a major environmental challenge [49]. These studies are crucial to address practical issues such as the localization of the deposited metal-containing dust in soil cover to prevent the migration of pollutants to ecosystems. Additionally, under field conditions, these studies are complicated due to the high level of uncertainty (element input to the experimental plots due to aerial emissions, soil erosion, lateral and vertical migration, and soil mixing by meso- and macrofauna). In temperate and subarctic climates with long-lasting snow cover, the input of pollutants to the soil is uneven (from spring to fall), which can also affect the geochemical behavior of elements.

## 5. Conclusions

Serpentine-containing materials obtained from the overburden rocks of a phlogopite deposit were used for the remediation of industrially polluted peat soil under conditions of ongoing aerial emissions in the vicinity of a Cu/Ni smelter in a subarctic region. Sequential soil treatment with 0.001, 0.01, 0.1, and 1.0 N HCl solutions allowed for the separation of element fractions with different geochemical mobilities, the estimation of the effect of the addition of serpentine materials on the migration of environmental toxicants, and the drawing of a hypothesis about the nature of the changes in this mobility.

In the series with both pyroxene and vermiculite–lizardite materials, the proportion of the copper fraction, which is capable of being displaced in phases from metal sorbents (i.e., organic matter and Fe/Mn oxyhydroxides), slightly increased. The results of NMDS data analysis demonstrated that the main factor causing the decrease in short-term mobility was the increase in pH as a result of the release of calcium and magnesium compounds into the soil solution. This fraction was deposited on the surface of mineral particles on an alkaline barrier. Thus, a decrease in the proportion of copper in the inert fraction and an increase in the potentially mobile fraction was observed. Additionally, the copper content in the readily mobile fraction did not considerably increase, which is most dangerous from the point of view of migration into the environment. Nickel and iron are typical isomorphic impurities of serpentine minerals; their inclusion in the structure of serpentines causes a decrease in their short-term and potential geochemical mobility.

Overall, the data show that fractionation using HCl solutions and the proposed coefficients of the short-term and potential geochemical migration of pollutants provides an adequate assessment of geochemical migration processes and can be used to assess the effectiveness of detoxification methods used for industrially polluted and degraded soils.

For practical use, all three serpentine-containing materials used in this study can be recommended. Notably, the procedure for increasing both the content and the activity of serpentine minerals as a result of thermal activation is impractical for the reclamation of large areas. A 25 vol.% serpentine-containing material in soil mixtures is sufficient to create a geochemical barrier with a stable, functioning vegetation cover.

## Figures and Tables

**Table 1:** Processing of the sequential fractionation results.

C(HCl), mol/L	Differential Fractions		Integral Fractions			Coefficients of Migration	
0.001	Cm1	Readily mobile	Csm	Short-term mobile	Cm1 + Cm2		
0.01	Cm2	Mobile					
0.1	Cp	Potentially mobile	Cps	Sum of mobile fractions	Csm + Cp	Km	Csm/Cps
1	Ca	Acid-soluble	Cct	Conditionally total	Cps + Ca		
			Cin	Inert	Cto-Cct	Kp	Cps/Cin
			Cto	Total		Ks	Cct/Cto

**Table 2:** Characteristics of studied materials.

Label	Material	Bulk Density, g/cm^3^	Moisture Capacity, wt.%	pH (H_2_O)	pH (KCl)
PR	Pyroxenite material	1.57	48	8.85	6.67
VL	Vermiculite–lizardite material	1.03	49	8.77	7.54
LT	Thermally activated vermiculite–lizardite material	0.65	90	9.72	9.54
PT	Industrially polluted peat soil	0.29	280	4.1	3.7

**Table 3:** Chemical composition of studied materials, wt.%.

Components	PT	PR	VL
SiO_2_	2.14	36.79	38.04
MgO	0.27	34.49	36.04
CaO	0.05	2.80	2.81
Al_2_O_3_	2.01	1.68	2.00
Fe_2_O_3_	3.77	10.5	5.71
MnO	0.03	0.34	0.18
TiO_2_	0.10	0.13	0.12
NiO	0.30	0.05	0.05
Cr_2_O_3_	0.02	0.04	0.03
P_2_O_5_	0.27	0.10	0.05
K_2_O	0.27	0.16	0.01
Cl	0.10	0.14	0.14
CuO	0.75	–	–
LOI	89.00	12.75	14.82

Note: LOI—loss on ignition at 1000 °C; PT—initial peat soil; PR—pyroxenite material; VL—vermiculite–lizardite material.

**Table 4:** Composition and characteristics of soil samples.

Label	Proportion of Serpentinite		Bulk Density, kg∙dm^−3^	pH (H_2_O)	Eh
	vol.%	wt.%			
PR25	25	65	0.87	6.8	275
PR50	50	85	1.07	6.9	240
PR75	75	94	1.42	7.4	248
PR100	100	100	1.69	6.6	268
VL25	25	50	0.57	5.2	251
VL50	50	78	0.96	5.4	302
VL75	75	92	1.03	5.6	282
VL100	100	100	1.23	6.9	261
LT1(1)	1	2.5	0.30	5.8	296
LT1(2)	1	2.5	0.30	5.1	268
LT5(1)	5	11	0.38	4.9	268
LT5(2)	5	11	0.38	5.2	271
LT10(1)	10	20	0.38	4.8	304
LT10(2)	10	20	0.38	4.6	278
PT(1)	0	0	0.29	3.9	239
PT(2)	0	0	0.29	4.3	320

Note: PT—initial peat soil; PR—pyroxenite material; VL—vermiculite–lizardite material, LT—thermally activated vermiculite–lizardite material; 1…100—percentage (vol.) of mineral material in mixture; (1), (2)—repetition number.

## Data Availability

Data are contained within the article.
